# Supplementary material for: In situ observations of an active MoS2 model hydrodesulfurization catalyst
Source: Nat Commun. 2019 Jun 11;10:2546. doi: 10.1038/s41467-019-10526-0 (PMC6560102; doi:10.1038/s41467-019-10526-0)
Supplement: Supplementary file 1 — Supplementary Information [file 41467_2019_10526_MOESM1_ESM.pdf]

Supplementary Information

In situ observations of an active MoS_2 model hydrodesulfurization catalyst

Mom *et al.*

Supplementary Note 1

The electrons that make up the tunneling current in scanning tunneling microscopy (STM) originate from states close to the Fermi level. As the local density of these states is usually not evenly distributed over the atoms of the sample, the appearance of structures in STM often does not directly reflect the atomic structure. The edges of MoS_2 islands present a particularly pronounced case of such a discrepancy. Hence, in order to interpret the STM images properly, it is necessary to compare them to calculations of the local density of states using density functional theory (DFT). STM images are usually modeled as contours of equal values of the local density of states (LDOS) around the Fermi level. The LDOS around the Fermi level is typically obtained by summation over all LDOS contributions at and around the Fermi level with a Gaussian weighting (Tersoff-Hamann method[1,2]). Because the width of the Gaussian is somewhat arbitrary, we have chosen instead to sum over the LDOS from the Fermi level to the applied sample bias (-0.3 V), which does not require any other parameters. The additional benefit of this method is that the states further from the Fermi level are taken into account more appropriately.

Supplementary Figure 1 shows simulated STM images of the 100%S, 50%S and 50%S-50%H structures. From Supplementary Figure 1a, it is clear that the LDOS at the edge of the particle strongly deviates from the atomic arrangement for the 100%S structure. Hence, in the STM images for this structure the edge S atoms will appear not to follow the lattice of the particles' basal plane, even though in reality they do. The 50%S structure shows a similar discrepancy between atomic structure and LDOS (see Supplementary Figure 1b). However, a small fraction of the LDOS remains located around the S edge atoms, resulting in the red protrusions in the image. For a tip with a finite size however, the bright protrusions will dominate the image. Hence, the edge atoms will appear to be in registry with the basal plane S atoms.

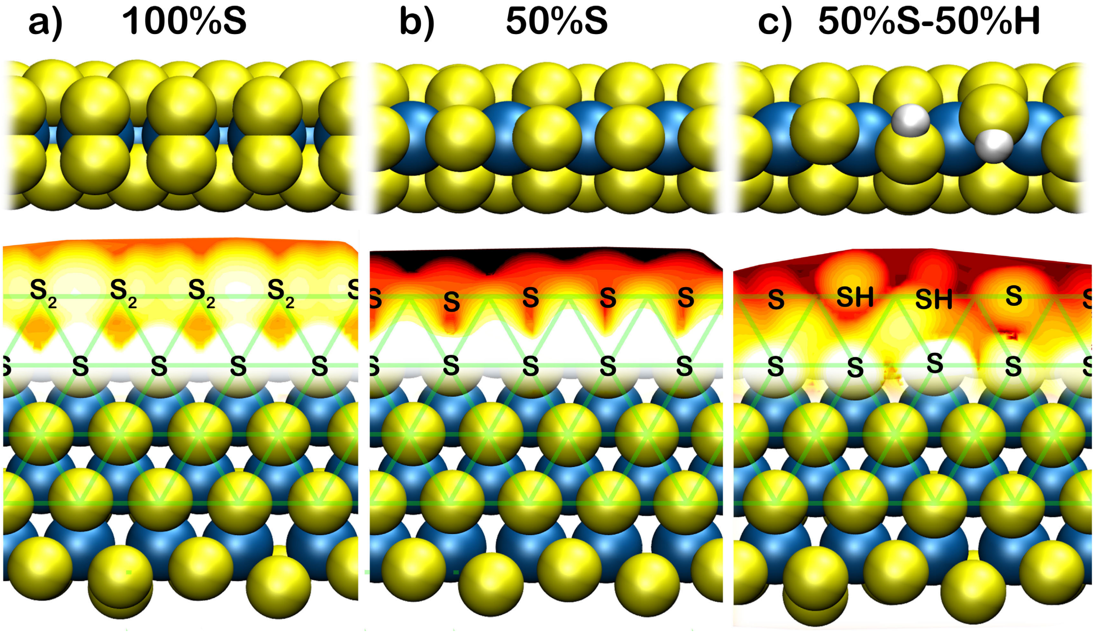

Supplementary Figure 1: Simulated STM images of a) the 100%S structure, b) the 50%S structure and c) the 50%S-50%H structure, for U_s = -0.3 V and at a countour value of $1x10^{-5}$ AU. The green grid facilitates comparison of the registry of the edge protrusions with respect to the basal plane S atoms.

For the case of the 50%S-50%H structure in Supplementary Figure 1c, the situation is less clear. Some of the protrusions on the edge are in registry with the basal plane, while others are not. At elevated temperatures, the H-atoms on the edge will diffuse at high rates, because the diffusion barrier is only 0.56 eV (obtained for unsupported MoS_2). To take this effect into account, we have averaged the LDOS over the four edge positions, as shown in Supplementary Figure 2. We see that the averaged structure has a similar appearance to that of the 50%S structure, although the protrusions that appear in registry with the basal plane atoms are less pronounced, whereras the out-of-registry protrusions at the outer edge are clearer. Therefore, the 50%S-50%H structure will appear similar to the 50%S structure, although with significantly less corrugation along the edge.

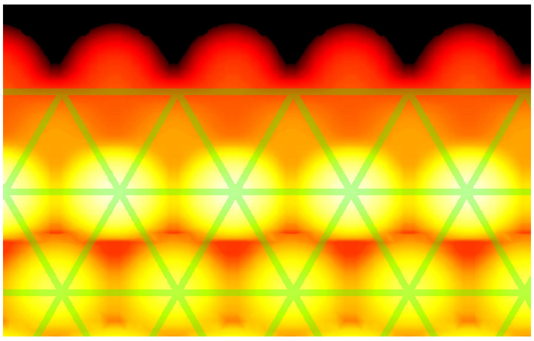

Supplementary Figure 2: Simulated STM image of the 50%S-50%H structure, taking into account thermal averaging over the edge positions, for U_s = -0.3 V and at a countour value of $1x10^{-5}$ AU. The green grid facilitates comparison of the registry of the edge protrusions with respect to the basal plane S atoms.

As noted in the main text, Bruix *et al.* obtained a different result for the appearance of the 50%S edge[3]. We identify two possible explanations. First, they used the Tersoff-Hamann method for the calculation of the LDOS around the Fermi level. As pointed out by Bollinger *et al.*, who found a result in agreement with ours, the choice of the Gaussian width is very sensitive in the particular case of MoS_2 due to its semiconducting properties[2]. Hence, the discrepancy between the different authors may be explained due to a different choice for this parameter. A second explanation may be a different placement of MoS_2 on the Au support. If we remove the gold in our calculations, the registry shift in the 50%S structure vanishes. Hence, the details of the MoS_2-Au interaction may be decisive in the obtained results.

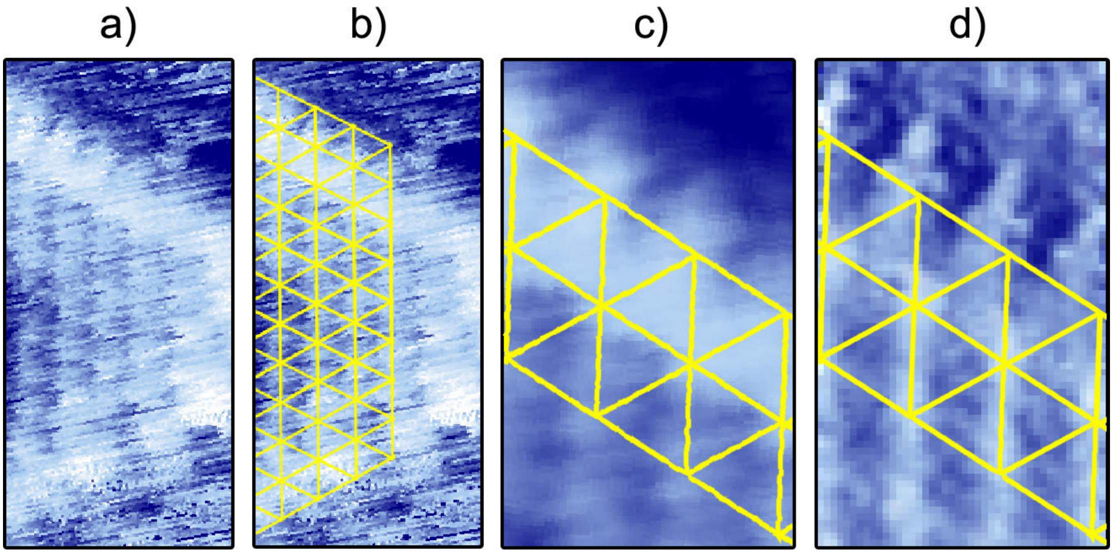

Supplementary Figure 3: Confirmation of the analysis of edge structures using derivative images. The image shown here corresponds to the same data as Figure 2a in the main text, obtained after preparation of MoS_2 in $2x10^{-6}$ mbar H_2S at 450 °C, imaged in UHV at room temperature. 6.6 x 6.6 nm^2, U_{bias} = -0.3 V, I_t = 560 pA. a) Height data, without any averaging. b) Same as a), with grid overlay. c) Averaged height data. d) Averaged derivative (in fast scanning direction) of height data, as also shown in Figure 2c in the main text.

For the appearance of edge structures in the experimental STM images, we excluded influences from the averaging and derivation procedure on the relative positions of edge and basal plane atoms (see Supplementary Figure 3). However, the tip shape may play a role. As can be seen in Figure 2 in the main text and Supplementary Figures 3-6, not all edges are depicted with equal sharpness. This is the result of an asymmetric tip shape (provided that the STM feedback was well-tuned, which was confirmed by the similar appearance of the left-to-right scanning and right-to-left scanning images). To confirm that the asymmetry in sharpness did not lead to misinterpretation of the edge structure in 1 bar H_2, we analyzed additional edges, as shown in Supplementary Figure 4. Again, we find that the edge protrusions appear in registry with the basal plane atoms. For HDS conditions, we analyzed a second edge of the same particle as shown in Figure 2 in the main text. As shown in Supplementary Figure 5, we again find that the edge protrusions are out of the registry of the basal plane atoms, confirming our analysis. Note that the overlay grids in Supplementary Figure 5 and Figure 2c in the main text were placed based on the alignment of a larger grid matching the basal plane lattice, the particle's corner, and both edges at the same time (see Supplementary Figure 6).

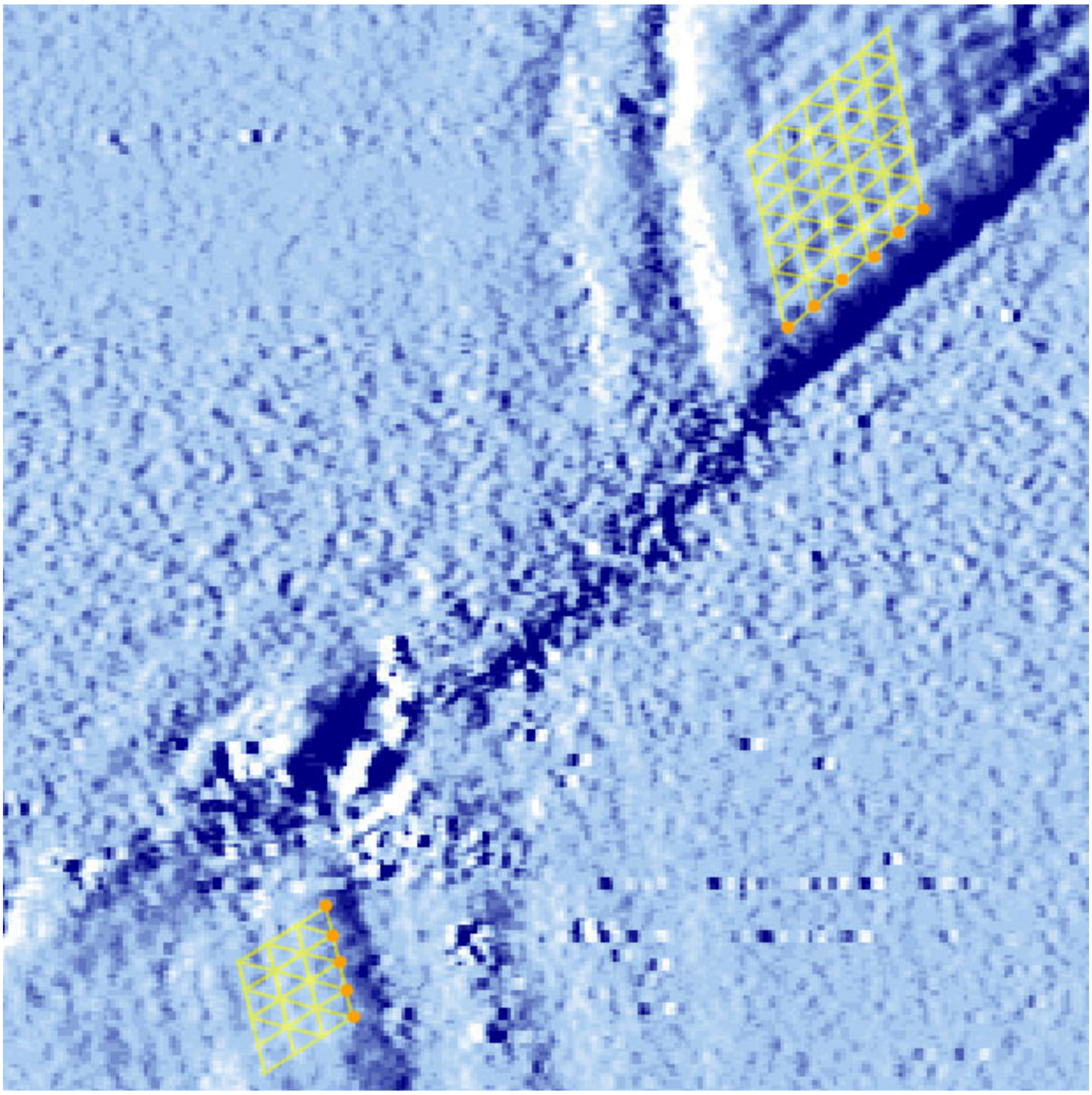

Supplementary Figure 4: MoS_2 edge structure in 1 bar H_2 at 250 °C. 10 x 10 nm², U_{bias} = -0.3 V

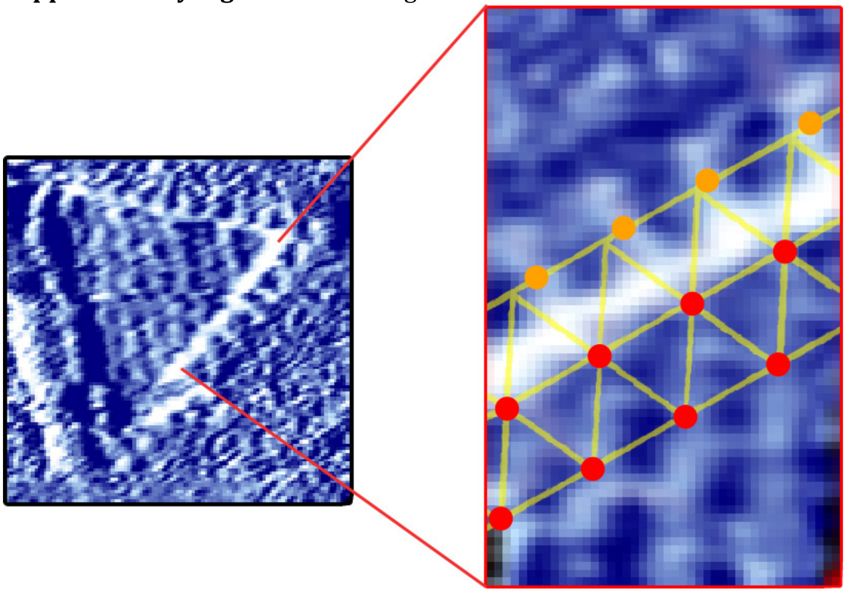

Supplementary Figure 5: Averaged edge unit cell during desulfurization of CH_3SH in 1 bar 1:9 CH_3SH/H_2 at 250 °C. 8 x 8 nm², U_{bias} = -0.3 V, I_t = 400 pA, based on the right edge of the same particle that was used in main text Figure 2c.

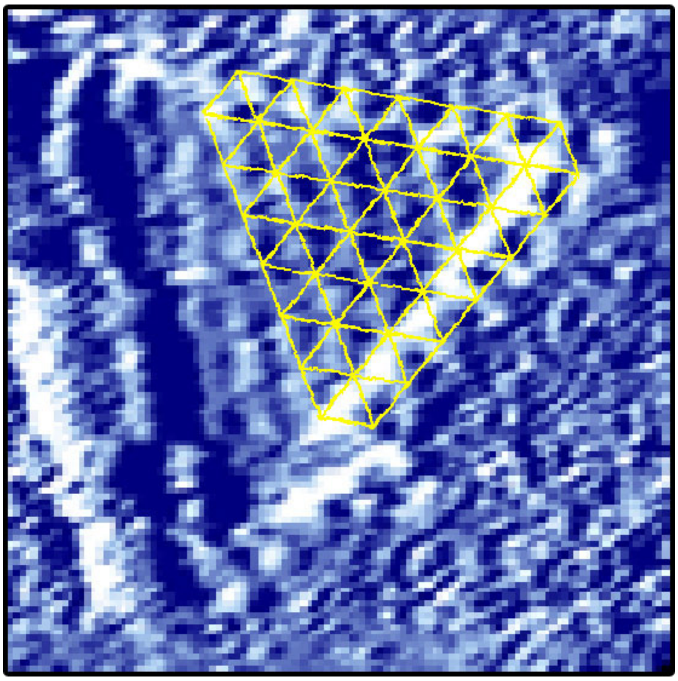

Supplementary Figure 6: Grid used for determination of the grid overlays in Supplementary Figure 4 and Figure 2c (main text).

Similar to literature observations[4,5], the reduced MoS_2 particles in 1 bar H_2 were observed to display bright edges (Supplementary Figure 7). While not very clear in the differentiated representation in Figure 2 in the main text, Supplementary Figure 7 shows that in the more traditional line-by-line background subtracted representation the particle's edges appear much brighter than the basal plane. This is in good agreement with our DFT studies, which indicate that this effect occurs for all the investigated edge structures. The bright appearance of the edges originates from metallic states around the Fermi level that are localized in particular at the edge of the particles, thus creating a high LDOS around the edges.

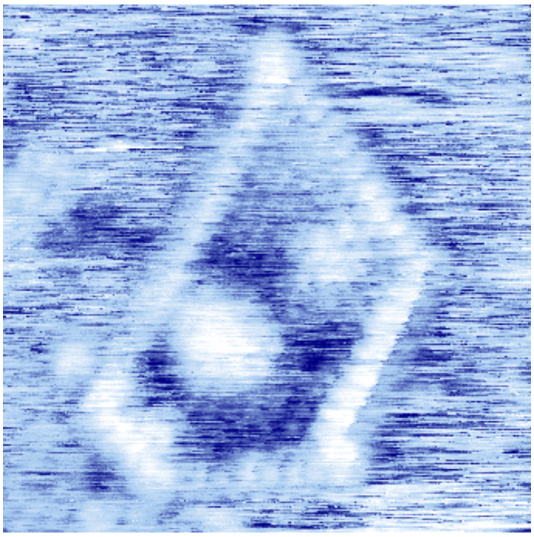

Supplementary Figure 7: Same image as shown in Figure 2 in the main text, shown as a line-by-line background subtracted image to clearly represent high LDOS at the particle edges. Catalyst imaged in 1 bar H_2 at 50 °C. 6.6 x 6.6 nm^2, U_{bias} = -0.3 V, I_t = 630 pA

Supplementary Note 2

To assess the influence of the Au(111) support on the relative stability of MoS_2 edge structures, phase diagrams were computed with and without Au(111) support, as shown in Supplementary Figure 8. Clearly, gold-supported MoS_2 favors higher edge sulfur coverage than its unsupported counterpart. Nonetheless, Au(111) is considered a weakly interacting support[6] since it does not significantly change the edge structures. In contrast, oxide supports like TiO_2 and Al_2O_3 are thought to make oxygen linkages at the particle edges[6–8].

We should point out that the effect of the Au support may be overestimated in our calculations, as the Au(111) substrate was stretched to match the MoS_2 lattice. Such a stretched surface tends to be more reactive[9], resulting in a more pronounced support effect. Some of this stretching also occurs experimentally, lifting the Au(111) herringbone reconstruction underneath and in the direct vicinity of the MoS_2 particles[10]. However, the moiré pattern observed on larger MoS_2 particles[11] shows that there is still a lattice mismatch between Au(111) and MoS_2. Hence, we expect some overestimation in the effect of the Au support in our calculations, which implies a slight overestimation of the stability of higher-sulfur-coverage structures.

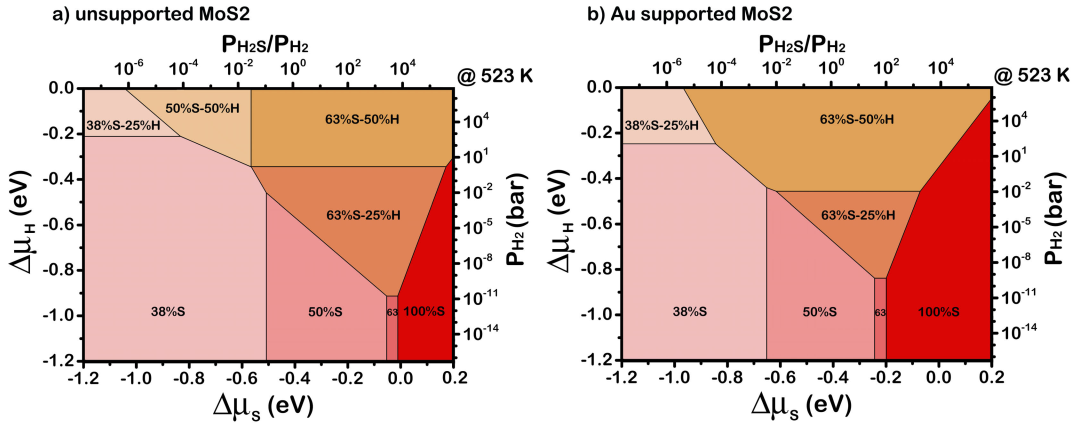

Supplementary Figure 8: *Ab initio* thermodynamics phase diagrams of the MoS_2 edge structure in H_2/H_2S mixtures. a) Unsupported MoS_2. b) Au-supported MoS_2.

Supplementary Note 3

To establish the validity of our calculations, we compared our results to those reported in the literature. We note that in previously published phase diagrams, the scale for the sulfur chemical potential was defined in different ways, leading to offsets with respect to our scale. Bollinger *et al.*[2], Lauritsen *et al.*[4] and Prodhomme *et al.*[12] referenced their scale against bulk sulfur in its alfa phase. This leads to an offset of $E_{H2S} - E_{H2} - E_{S\alpha}$ with respect to our $\Delta\mu_S$ scale. Based on the conversion to pressure (ratio) at specific temperatures mentioned in the respective articles, we estimate this offset to be -0.1 eV for Bollinger *et al.*[2] and Lauritsen *et al.*[4] and -0.33 eV for Prodhomme *et al.*[12]. In the case of Cristol *et al.*, an offset of $H_{H2S} - H_{H2}$ leads to a shift of -4.15 eV with respect to our scale (again calculated using the combinations of pressure, temperature and chemical

potential mentioned in the text). Using these offsets, we constructed the comparison in Supplementary Table 1. From these results, it is clear that the relative energies of various edge structures are reproduced relatively well, independent of the differences in methods.

We should note that there were also differences in the employed unit cells and the range of investigated structures. Where Bollinger *et al.* and Lauritsen *et al.* used a 2-Mo-atom wide unit cell, Cristol *et al.* employed a 3-Mo-atom wide unit cell and Prodhomme *et al.* a 4-Mo-atom wide cell. Obviously, larger unit cells lead to more flexibility in the observed structures. The low-symmetry 38%S-x%H and 63%S-x%H structures which we have found to be stable over a wide range of conditions were not accessable to Bollinger *et al.*, Lauritsen *et al.* and Cristol *et al.*. Prodhomme *et al.* did use the same unit cell size as the present work, but did not consider structures with a coverage higher than 50%S. On the other hand, they did consider the 38%S structures and found their stability somewhat lower than our work: the transition from 38%S to 50%S occurs already at $\Delta\mu_S$=-0.76 eV, whereas our calculations (without Au support) put the transition at -0.51 eV. This difference could be related to the differences in used methodology (VASP/projector augmented waves for Prodhomme *et al.* versus the orbital-based basis set used in the BAND package employed here). The orbital-based basis set used in our case is particularly suited for the simulation of situations where the electron density shows strong gradients, as is the case with the irregular 38%S-x%H and 63%S-x%H structures. This could lead to a higher stabilization of these structures.

Supplementary Table 1: Relative stability of 50%S and 100%S structures

Authors	$\Delta\mu_{H\text{-}50S/50S\text{-}50H}$ unsupported	$\Delta\mu_{H\text{-}50S/50S\text{-}50H}$ Au supported	$\Delta\mu_{S\text{-}50S/100S}$ unsupported	$\Delta\mu_{S\text{-}50S/100S}$ Au supported
Present	-0.37 eV	-0.30 eV	-0.02 eV	-0.21 eV
Bollinger *et al.*[2]	-0.30 eV		-0.11 eV	
Lauritsen *et al.*[4]		-0.30 eV		-0.33 eV
Prodhomme *et al.*[12]	-0.35 eV			
Cristol *et al.*[13]			0.01 eV	

Supplementary Note 4

The adsorption energy of CH_3SH on Au-supported MoS_2 was calculated for a coverage of 1 molecule per 4 Mo edge atoms. For the calculation of the free energy of adsorption, entropic contributions were included using the reaction conditions in the STM experiment (P_{CH3SH} = 0.1 bar, P_{H2S} = ~0.001 bar, P_{H2} = 0.9 bar, 523 K). The formation energy of the adsorption structures was referenced against the most stable structure in the absence of CH_3SH, namely 63%S-50%H.

Supplementary Table 2: Energetics of CH_3SH adsorption on Au-supported MoS_2

Structure	E_{ads}	ΔG_{ads}	ΔG_{form}
38%S	-1.62 eV	-0.13 eV	-0.08 eV
50%S-50%H	-1.28 eV	0.21 eV	0.44 eV
87%S	-0.46 eV	1.03 eV	2.90 eV

Similar to the situation for sulfur deposition in the previous section, CH_3SH adsorption is stabilized by the Au support: the adsorption energies in the unsupported case are -1.23 eV for 38%S, -1.14 eV for 50%S-50%H and -0.30 eV for 87%S. Note that these values are in good agreement with the literature (-1.12 eV for 38%S[14]) and with the infrared spectroscopy identifying only weak adsorption of CH_3SH on stongly sulfided MoS_2[15].

Supplementary Note 5

The coverage of different edge structures under steady state conditions were modeled using the reaction network shown in Supplementary Figure 9.

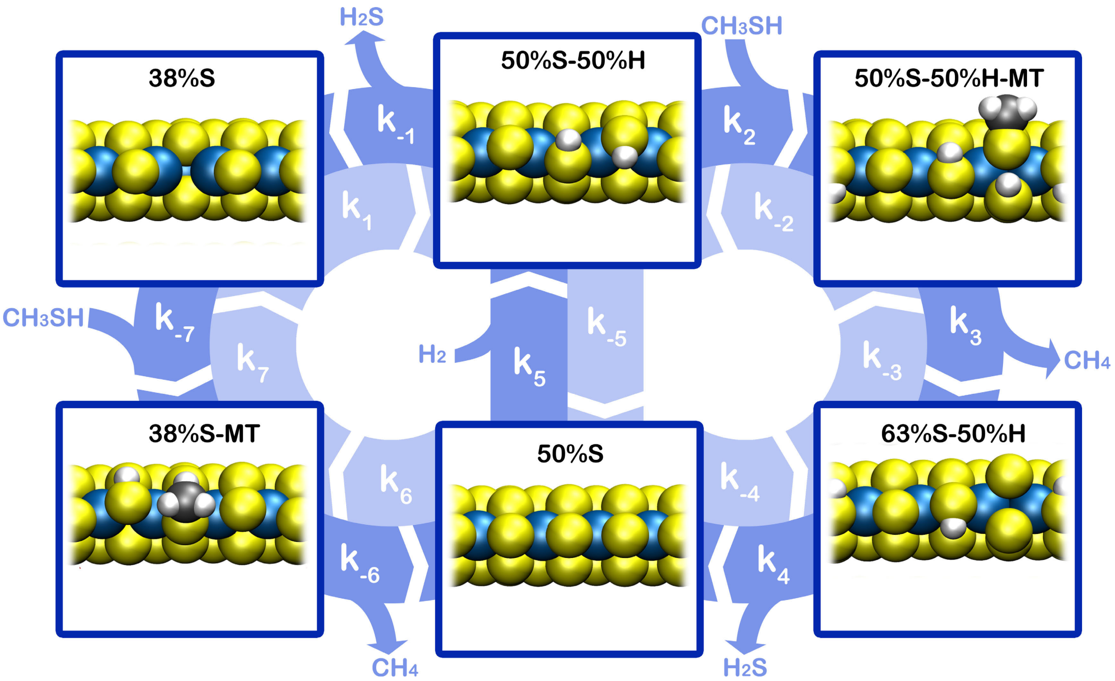

Supplementary Figure 9: Reaction network for CH_3SH desulfurization on MoS_2/Au(111). The rate constants k_i are used in Supplementary Equations 2-7 detailed below. “MT” corresponds to methane thiol.

It is assumed that only 6 different edge states exist:

$$\theta_{38} + \theta_{50-50} + \theta_{50-50MT} + \theta_{63-50} + \theta_{50} + \theta_{38MT} = 1 \quad (1)$$

As shown in Supplementary Figure 7, each edge state defines the S, H and CH_3SH coverage per block of 4 Mo edge atoms. It is assumed that there is no correlation between adjacent 4-Mo blocks, allowing us to establish global rate equations. Assuming steady state conditions, we can write:

$$\frac{d\theta_{38}}{dt} = k_{-1}\theta_{50-50} + k_7\theta_{38MT} - k_1\frac{P_{H_2S}}{P^o}\theta_{38} - k_{-7}\frac{P_{MT}}{P^o}\theta_{38} = 0 \quad (2)$$

$$\frac{d\theta_{50-50}}{dt} = k_1\frac{P_{H_2S}}{P^o}\theta_{38} + k_5\frac{P_{H_2}}{P^o}\theta_{50} + k_{-2}\theta_{50-50MT} - \left(k_{-1} + k_{-5} + k_2\frac{P_{MT}}{P^o}\right)\theta_{50-50} = 0 \quad (3)$$

$$\frac{d\theta_{50-50MT}}{dt} = k_2 \frac{P_{MT}}{P^o} \theta_{50-50} + k_{-3} \frac{P_{CH_4}}{P^o} \theta_{63-50} - k_{-2}\theta_{50-50MT} - k_3\theta_{50-50MT} = 0 \quad (4)$$

$$\frac{d\theta_{63-50}}{dt} = k_3\, \theta_{50-50MT} + k_{-4} \frac{P_{H_2S}}{P^o} \theta_{50} - k_{-3} \frac{P_{CH_4}}{P^o} \theta_{63-50} - k_4\, \theta_{63-50} = 0 \quad (5)$$

$$\frac{d\theta_{50}}{dt} = k_4\, \theta_{63-50} + k_{-5}\, \theta_{50-50} + k_{-6}\theta_{38MT} - \left(k_{-4} \frac{P_{H_2S}}{P^o} + k_5 \frac{P_{H_2}}{P^o} + k_6 \frac{P_{CH_4}}{P^o}\right) \theta_{50} = 0 \quad (6)$$

$$\frac{d\theta_{38MT}}{dt} = k_6 \frac{P_{CH_4}}{P^o} \theta_{50} + k_{-7} \frac{P_{MT}}{P^o} \theta_{38} - k_{-6}\, \theta_{38MT} - k_7\, \theta_{38MT} = 0 \quad (7)$$

The rate constants (k_i) in these equations were calculated from the free energy barriers ($\Delta G^0_{a\_i}$) using transition state theory:

$$k = \frac{k_B T}{h}\, e^{\frac{-\Delta G^0_a}{k_B T}} \quad (8)$$

The standard pressure P_0 in Supplementary Equations 2-7 is the pressure for which $\Delta G^0_{a\_i}$ is defined (1 bar here). By inserting the experimental temperature and pressures, the set of linear equations (Supplementary Equations 1-7) becomes solvable using matrix diagonalization, yielding the steady state coverage for each of the 6 investigated edge structures.

Supplementary References

1. Tersoff, J. & Hamann, D. R. Theory of the scanning tunneling microscope. *Phys. Rev. B* **31,** 805–813 (1985).
2. Bollinger, M. V., Jacobsen, K. W. & Nørskov, J. K. Atomic and electronic structure of MoS2 nanoparticles. *Phys. Rev. B* **67,** 085410 (2003).
3. Bruix, A. *et al.* In Situ Detection of Active Edge Sites in Single-Layer MoS 2 Catalysts. *ACS Nano* **9,** 9322–9330 (2015).
4. Lauritsen, J. V. *et al.* Atomic-scale insight into structure and morphology changes of MoS2 nanoclusters in hydrotreating catalysts. *J. Catal.* **221,** 510–522 (2004).
5. Füchtbauer, H. G. *et al.* Morphology and atomic-scale structure of MoS2 nanoclusters synthesized with different sulfiding agents. *Top. Catal.* **57,** 207–214 (2014).
6. Walton, A. S., Lauritsen, J. V., Topsøe, H. & Besenbacher, F. MoS2 nanoparticle morphologies in hydrodesulfurization catalysis studied by scanning tunneling microscopy. *J. Catal.* **308,** 306–318 (2013).
7. Hinnemann, B., Nørskov, J. K. & Topsøe, H. A density functional study of the chemical differences between Type I and Type II MoS2-based structures in hydrotreating catalysts. *J. Phys. Chem. B* **109,** 2245–53 (2005).
8. Topsøe, N. & Topsøe, H. FTIR studies of Mo/Al2O3 based catalysis. I.Morphology and Structure of Calcined and Sulfided Catalysts. *Journal of Catalysis* **139,** 631 – 640 (1993).
9. Mavrikakis, M., Hammer, B. & Nørskov, J. K. Effect of Strain on the Reactivity of Metal Surfaces. *Phys. Rev. Lett.* **81,** 2819–2822 (1998).
10. Lauritsen, J. V. *et al.* Hydrodesulfurization reaction pathways on MoS2

nanoclusters revealed by scanning tunneling microscopy. *J. Catal.* **224,** 94–106 (2004).

11. Krane, N., Lotze, C. & Franke, K. J. Moiré structure of MoS2 on Au (111): Local structural and electronic properties. *Surf. Sci.* (2018). doi:10.1016/j.susc.2018.03.015
12. Prodhomme, P. Y., Raybaud, P. & Toulhoat, H. Free-energy profiles along reduction pathways of MoS2 M-edge and S-edge by dihydrogen: A first-principles study. *J. Catal.* **280,** 178–195 (2011).
13. Cristol, S. *et al.* Theoretical Study of the MoS2(100) Surface: A Chemical Potential Analysis of Sulfur and Hydrogen Coverage. *J. Phys. Chem. B* **106,** 5659–5667 (2002).
14. Joshi, Y. V., Ghosh, P., Venkataraman, P. S., Delgass, W. N. & Thomson, K. T. Electronic descriptors for the adsorption energies of sulfur-containing molecules on co/mos2, using dft calculations. *J. Phys. Chem. C* **113,** 9698–9709 (2009).
15. Maugé, F., Lamotte, J., Nesterenko, N. S., Manoilova, O. & Tsyganenko, A. A. FT-IR study of surface properties of unsupported MoS2. *Catal. Today* **70,** 271–284 (2001).
