# Supplementary material for: In situ observations of an active MoS2 model hydrodesulfurization catalyst
Source: Nat Commun. 2019 Jun 11;10:2546. doi: 10.1038/s41467-019-10526-0 (PMC6560102; doi:10.1038/s41467-019-10526-0)
Supplement: Supplementary file 3 — Description of Additional Supplementary Files [file 41467_2019_10526_MOESM3_ESM.pdf]

Description of Additional Supplementary Files

File Name: Supplementary Data 1
Description: STM data Figure 1a

File Name: Supplementary Data 2
Description: STM data Figure 1b

File Name: Supplementary Data 3
Description: STM data Figure 1c

File Name: Supplementary Data 4
Description: STM data Figure 2a

File Name: Supplementary Data 5
Description: STM data Figure 2b

File Name: Supplementary Data 6
Description: STM data Figure 2c

File Name: Supplementary Data 7
Description: DFT energies for the calculated structures

File Name: Supplementary Data 8
Description: LDOS summed between the Fermi level and to the applied sample bias (--0.3V) for the 100%S Mo edge

File Name: Supplementary Data 9
Description: LDOS summed between the Fermi level and to the applied sample bias (--0.3V) for the 50%S Mo edge

File Name: Supplementary Data 10
Description: LDOS summed between the Fermi level and to the applied sample bias (--0.3V) for the 50%S Mo edge, with larger box

File Name: Supplementary Data 11
Description: LDOS summed between the Fermi level and to the applied sample bias (--0.3V) for the 50%S,50%H Mo edge

File Name: Supplementary Data 12
Description: LDOS summed between the Fermi level and to the applied sample bias (--0.3V) for the 50%S,50%H Mo edge, larger box

File Name: Supplementary Data 13
Description: LDOS summed between the Fermi level and to the applied sample bias (--0.3V) for the 38%S-MT Mo edge
