# Supplementary figures and images for: In situ observations of an active MoS2 model hydrodesulfurization catalyst
Source: Nat Commun. 2019 Jun 11;10:2546. doi: 10.1038/s41467-019-10526-0 (PMC6560102; doi:10.1038/s41467-019-10526-0)

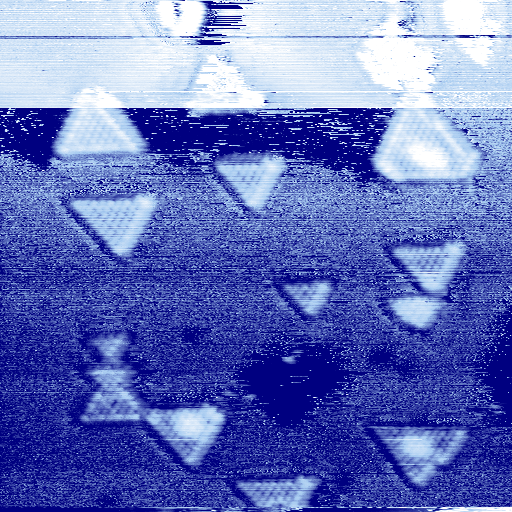

Supplement: Supplementary file 4 — Supplementary Data 1 [file 41467_2019_10526_MOESM4_ESM.bmp]

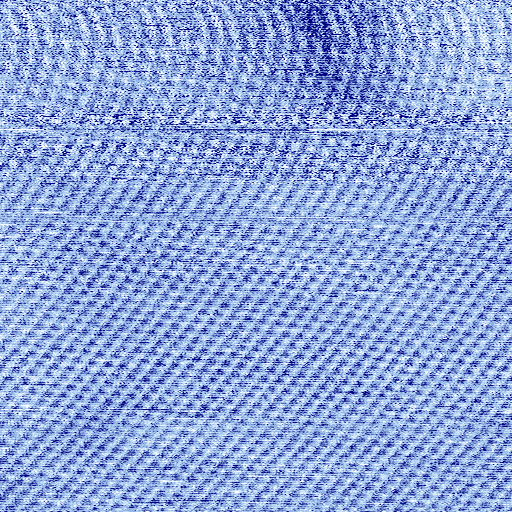

Supplement: Supplementary file 5 — Supplementary Data 2 [file 41467_2019_10526_MOESM5_ESM.bmp]

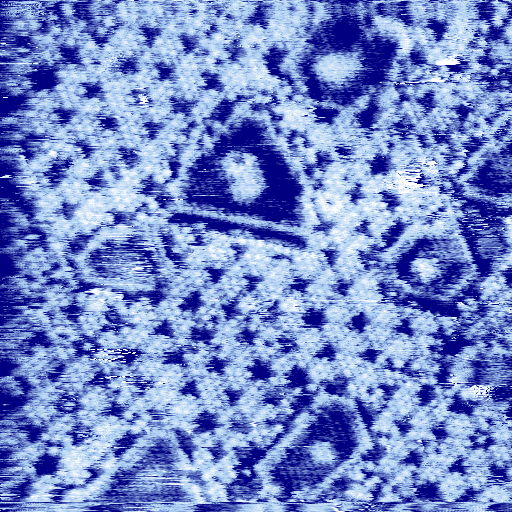

Supplement: Supplementary file 6 — Supplementary Data 3 [file 41467_2019_10526_MOESM6_ESM.bmp]

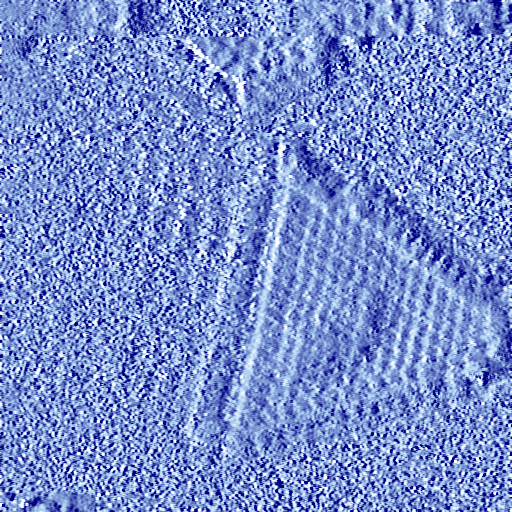

Supplement: Supplementary file 7 — Supplementary Data 4 [file 41467_2019_10526_MOESM7_ESM.png]

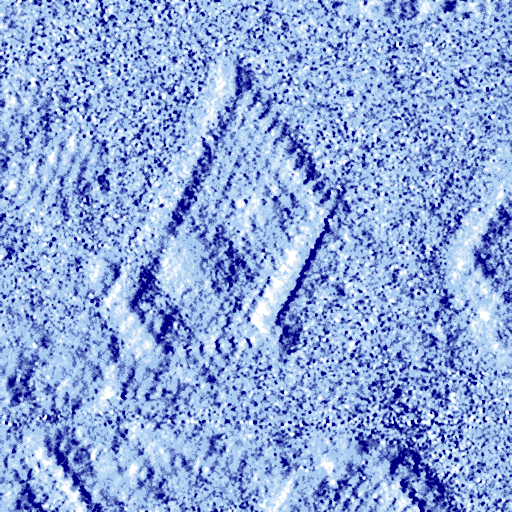

Supplement: Supplementary file 8 — Supplementary Data 5 [file 41467_2019_10526_MOESM8_ESM.png]

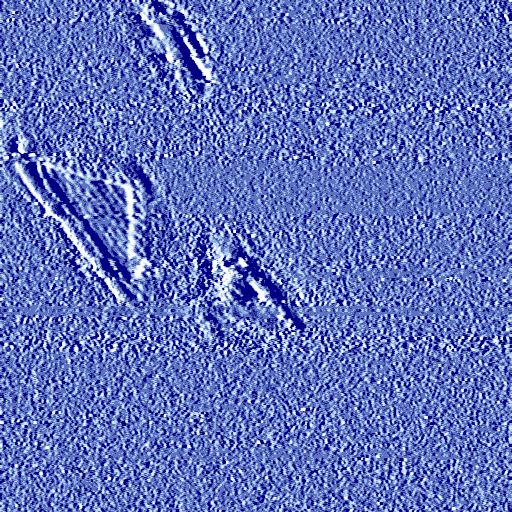

Supplement: Supplementary file 9 — Supplementary Data 6 [file 41467_2019_10526_MOESM9_ESM.png]
